# Aspects of Molecular Genetics in Dromedary Camel

**DOI:** 10.3389/fgene.2021.723181

**Published:** 2021-10-21

**Authors:** Mohammed Piro

**Affiliations:** Veterinary Genetics Laboratory (LAGEV), Hassan II Agronomic and Veterinary Institute, Rabat, Morocco

**Keywords:** molecular genetics, microsatellite, SNP, mtDNA, dromedary camel

## Abstract

Dromedary camels are unique in their morphological and physiological characteristics and are capable of providing milk and meat even under extreme environmental conditions. Like other species, the dromedary camel has also benefitted from the development of the molecular genetics to increase the knowledge about different aspect in camel genetics (genetic variation, molecular marker, parentage control, gene of interest, whole genome, dating…etc.). In this paper we review the different molecular genetic technics used in this particular species and future prospects. Dromedary genetic studies started in the end of the 1980s with phenotypic evaluation and the attempts to highlight the protein and biochemical diversity. In the 2000s, with the development of molecular markers such as microsatellites, genetic diversity of different types in several countries were estimated and microsatellites were also used for parentage control. In terms of genetic characterization, microsatellites revealed a defined global structure, differentiating East African and South Arabian dromedaries from North African, North Arabian, and South Asian individuals, respectively. Also, mitochondrialDNA sequence analysis of ancient DNA proved to be crucial in resolving domestication processes in dromedaries. Ancient and modern DNA revealed dynamics of domestication and cross-continental dispersion of the dromedary. Nuclear SNPs, single nucleotide polymorphisms changes that occur approximately each 1000 bps in the mammalian genome were also applied in some studies in dromedary. These markers are a very useful alternative to microsatellites and have been employed in some studies on genetic diversity and relevant phenotypic traits in livestock. Finally, thanks to the use of Next Generation Sequencing (NGS) the whole-genome assemblies of the dromedary (*Camelus dromedarius*) and a work to establish the organization of the dromedary genome at chromosome level were recently published.

## Introduction

Genetic characterization and assessment of genetic diversity is the primary step in the conservation and the management of genetic resources (Rout et al., 2008). Genetic characterization and diversity can be assessed within and between populations by different methods including biochemical and molecular techniques.

Like other species, the dromedary camel has also benefited from the development of the molecular genetics to improve our knowledge about different aspects in camel genetics (genetic variation, molecular markers, parentage control, gene of interest, whole genome, dating….). Old World camelids have a specific morphological and physiological characteristic and can provide milk and meat even under extreme harsh conditions. Dromedary camels first appeared was estimated in the middle Eocene (around 40 million years ago) and the first ancestors of the camelid’s family were found in North America (Balmus et al., 2007). Afterward, they split into Old and New world camelids. The old-world camelids migrated to the eastern hemisphere and differentiated into two species: *Camelus bactrianus* and *Camelus dromedarius*. The split between Old and New world camelids was estimated by using molecular studies to have happened 11–16 million years ago (Kadwell et al., 2001) or 25 million years ago (Ji et al., 2009). The divergence between one-humped (*Camelus dromedarius*) and two-humped (*Camelus bactrianus*) camel species was estimated between 4.4 and 8 million years ago (Ji et al., 2009; Wu et al., 2014). The wild two humped camels (*Camelus ferus*) has been recently recognized as a separate species, based on molecular genetic data especially by mitochondrial DNA and nuclear markers and the time of separation was estimated around 0.6 and 1.8 million years ago (Mohandesan et al., 2017).

Investigations on Dromedary genetics started in the end of the 1980s with phenotypic evaluation and the attempt to highlight the protein and biochemical diversities. In the 2000s, with the development of molecular markers such as microsatellites, genetic diversity of different types or breeds of dromedary camels was estimated in several countries (Mburu et al., 2003) and microsatellites were also used for parentage control (Mariasegaram et al., 2002). Also, comprehension of domestication process was resolved using mtDNA sequence analysis of ancient DNA (Almathen et al., 2016). Ancient and modern DNA revealed dynamics of domestication and cross-continental dispersion of the dromedary camel (Almathen et al., 2016; Ciani, 2018). Nuclear SNPs, single base pair changes that occur approximately each 1,000 bps in the mammalian genome are also applied in some studies in the dromedary camel (Sushma et al., 2014; Abd El-Aziem et al., 2015; Ruiz et al., 2015; Lado et al., 2020a). These markers can be used as an alternative to microsatellites especially in genetic diversity studies and detection of relevant phenotypic traits in livestock.

With the implementation of the Next Generation Sequencing (NGS) technique, two whole-genome assemblies for the camel (*Camelus dromedarius*) were published (GenBank assembly accession: GCA_000803125.1) (Fitak et al., 2015) with the reference genome for the wild camel (*Camelus ferus*) (GenBank assembly accession: GCA_000311805.2) (Wu et al., 2014) and two whole-genome assemblies for the Bactrian camel (*Camelus Bactrianus*) (GenBank assembly accession: GCA_000604445.1 (Burger and Palmeri, 2014) and GCA_000767855.1 (Wu et al., 2014); while, another independent whole genome assembly was established in 2016. Recently, studies established the organization of the dromedary genome at the chromosome level (Fitak et al., 2015; Ruvinskiy et al., 2019; Lado et al., 2020a; Ming et al., 2020).

## Dromedary Camel’s Genetic Markers

Several genetic markers such as microsatellites, mtDNA and SNP can be used for genetic characterization of different species or breeds, parentage control and/or determination of traits of economic interest by genome wild associations studies (GWAS), an essential step for their use for selection assisted by markers or introgression.

### Microsatellites

Microsatellites are used in studies aimed at characterizing genetic diversity studies because of their simple use and also the very low genetic variation in protein polymorphism (Guerouali and Acharbane, 2004). A large number of studies has been conducted, all around the world, on the genetic diversity of livestock species based on microsatellite loci. The number of loci to be genotyped or the size of the samples per breed is necessary to correctly analyzing the results. Studies in livestock describe a minimum of 8–10 loci (Cornuet et al., 1999; Arthofer et al., 2018) and more than 25 to 30 individuals per population for population genetic studies based on microsatellite allele frequencies. (Cornuet et al., 1999; Hale et al., 2012).

A set of Camelidae microsatellites was generated from published data on New World camelids: Alpacas and Ilamas (Obreque et al., 1998; Penedo et al., 1999), and several research studies have successfully assessed genetic variability in dromedary camels using these microsatellites (Jianlin et al., 2000; Sasse et al., 2000). This set comprises six-teen primers with highest polymorphism (VOLP03, VOLP08, VOLP10, VOLP32, VOLP67, YWLL02, YWLL08, YWLL09, YWLL38, YWLL44, LCA33, LCA37, LCA56, LCA63 LCA66, LCA77) (Nolte et al., 2005).

In 2002, Mariasegaram and collaborators were able to determine eight camel-specific microsatellites: CVRL01, CVRL02, CVRL03, CVRL04, CVRL05, CVRL06, CVRL07 and CVRL08.

The international panel on Animal Genetic Diversity (ISAG-FAO, 2004) recommended a list of 25 microsatellites markers for the evaluation of genetic diversity in camelids (Table 1), namely CMS09, CMS13, CMS15, CMS17, CMS18, CMS25, CMS32, CMS50, CMS121, CVRL01, CVRL02, CVRL05, CVRL06, CVRL07, LCA66, VOLP03, VOLP08, VOLP10, VOLP32, VOLP67, YWLL08, YWLL09, YWLL38, YWLL44 and YWLL59. Sixteen out of these 25 microsatellites are considered to be the most polymorphic and are thus highly recommended for dromedary camel genetic characterization. These are: YWLL08, YWLL09, YWLL38, YWLL44, YWLL59, VOLP03, VOLP08, VOLP10, VOLP32, VOLP67, LCA66, CVRL01, CVRL05, CVRL06, CVRL07 and CMS50 (Nolte et al., 2005).

**TABLE 1:** Primers, allele range and reference of the 25 microsatellites recommended for Dromedary camel by the International Panel on Animal Genetic Diversity.

Locus	Primers	Allele range	References
VOLP03	F-GCCAAAATAGGCTTACCCTTG	144–176	Obreque et al. (1998)
	R-CCCGCTTCATCTATTGGAAA		
VOLP08	F-CCATTCACCCCATCTCTC	142–180	Obreque et al. (1998)
	TCG​CCA​GTG​ACC​TTA​TTT​AGA		
VOLP10	F-CTTCTCCTTCCTCCCTACT	249–267	Obreque et al. (1998)
	R-CGTCCACTCCTTCATTC		
VOLP32	F-GTGATCGGAATGGCTTGAAA	192–262	Obreque et al. (1998)
	R-CAGCGAGCACCTGAAAGAA		
VOLP67	F-TTAGAGGGTCTATCCAGTTTC	149–203	Obreque et al. (1998)
	R-TGGACCTAAAAGAGTGGAG		
YWLL02	F-GTGCACTCAGATACCTTCACA	290–304	Lang et al. (1996)
	R-TACATCTGCAATGATCGACCC		
YWLL08	F-ATCAAGTTTGAGGTGCTTTCC	134–172	Lang et al. (1996)
	R-CCATGGCATTGTGTTGAAGAC		
YWLL09	F-AAGTCTAGGAACCGGAATGC	158–162	Lang et al. (1996)
	R-AGTCAATCTACACTCCTTGC		
YWLL38	F-GGCCTAAATCCTACTAGAC	174–192	Lang et al. (1996)
	R-CCTCTCACTCTTGTTCTCCTC		
YWLL44	F-CTCAACAATGCTAGACCTTGG	090–114	Lang et al. (1996)
	R-GAGAACACAGGCTGGTGAATA		
YWLL59	F-TGTGCAGGAGTTAGGTGTA	96–136	Lang et al. (1996)
	R-CCATGTCTCTGAAGCTCTGGA		
LCA66	F-GTGCAGCGTCCAAATAGTCA	212–262	Penedo et al. (1999)
	R-CCAGCATCGTCCAGTATTCA		
CMS9	F-TGCTTTAGACGACTTTTACTTTAC	227–256	Evdotchenko et al. (2003)
	R-ATTTCACTTTCTTCATACTTGTGAT		
CMS13	F-TAGCCTGACTCTATCCATTTCTC	238–265	Evdotchenko et al. (2003)
	R-ATTATTTGGAATTCAACTGTAAGG		
CMS15	F-AAAACTAAAGCCAGAAAGGCAAA	81–121	Evdotchenko et al. (2003)
	R-TTTTTCCAGATCTTGCACCAC		
CMS17	F-TATAAAGGATCACTGCCTTC	149–167	Evdotchenko et al. (2003)
	R-AAAATGAACCTCCATAAAGTTAG		
CMS18	F-GAACGACCCTTGAAGACGAA	144–166	Evdotchenko et al. (2003)
	R-AGCAGCTGGTTTTAGGTCCA		
CMS25	F-GATCCTCCTGCGTTCTTATT	93–102	Evdotchenko et al. (2003)
	R-CTAGCCTTTGATTGGAGCAT		
CMS32	F-ACGGACAAGAACTGCTCATA	198–209	Evdotchenko et al. (2003)
	R-ACAACCAATAAATCCCCATT		
CMS50	F-TTTATAGTCAGAGAGAGTGCTG	149–191	Evdotchenko et al. (2003)
	R-TGTAGGGTTCATTGTAACA		
CMS121	F-CAAGAGAACTGGTGAG GATTTTC	147–167	Evdotchenko et al. (2003)
	R-AGTTGATAAAAATACAGCTGGAAAG		
CVRL01	F-GAAGAGGTTGGGGCACTAC	188–253	Mariasegaram et al. (2002)
	R-CAGGCAGATATCCATTGAA		
CVRL02	F-TGTCACAAATGGCAAGAT	205–215	Mariasegaram et al. (2002)
	R-AGTGTACGTAGCAGCATTATTT		
CVRL05	F-CCTTGGACCTCCTTGCTCTG	148–174	Mariasegaram et al. (2002)
	R-GCCACTGGTCCCTGTCATT		
CVRL06	F-TTTTAAAAATTCTGACCAGGAGTCTG	185–205	Mariasegaram et al. (2002)
	R-CATAATAGCCAAAACATGGAAACAAC		
CVRL07	F-AATACCCTAGTTGAAGCTCTGTCCT	279–299	Mariasegaram et al. (2002)
	R-GAGTGCCTTTATAAATATGGGTCTG		

Furthermore, the different studies on dromedary genetic diversity were not limited to these 16 microsatellites, but used instead a range of different microsatellite markers whose the number and name of which differ from one study to another. Markers such as YWLL02, YWLL29, YWLL36, YWLL40, LCA18, LCA33 and CMS58, show a low amount of variability; whereas YWLL08, YWLL09, YWLL38, YWLL44, YWLL59, VOLP03, VOLP08, VOLP10, VOLP32, VOLP67, LCA66, CVRL01, CVRL05, CVRL06, CVRL07 and CMS50 show much more alleles per locus and also high PIC values. These markers are useful in describing heterozygosity levels and are more informative (Muneeb, 2014).

There is only one study, carried out by Sadder et al. (2015), that dealt with the development of simple sequence repeat (SSR) markers in four dromedary breeds using genome sequencing. These SSR markers can be used also in genetic studies of camels. Accordingly, the partial genome revealed 613 SSR loci with a minimum number of 5 repeat units. The SSR abundance was one in every 84.3 kb of contigs and the SSR loci comprised di- (80.8%), tri- (10.8%), tetra- (7.6%), and pentamer (0.8%) motifs (TA)n and (AC)n were the most abundant dimers (58.6%). Thirty SSR loci were experimentally characterized for dromedary and Bactrian camels.

### Mitochondrial DNA

The use of mitochondrial DNA (mtDNA) reflects the maternal inheritance and is useful for the establishment of genetic variation between species. For example, 1.9% nucleotide difference in the mitochondrial control regions (CR) was determined between Mongolian domestic and wild Bactrian camels (Ji et al., 2009; Silbermayr et al., 2010). On another hand, mtDNA sequence analysis showed the domestication processes in dromedaries and revealed the dynamics of domestication and cross-continental movement of the dromedary camel (Almathen et al., 2016). In this study, 1083 DNA samples from modern dromedaries were used. These samples were issued from 21 countries (Eastern Africa, Western and Northern Africa, North Arabian Peninsula, South Arabian Peninsula, and Southern Asia including samples from Australia). Seven DNA samples of early-domesticated dromedary specimens from Syria, Turkey, Jordan and Austria and eight wild dromedary specimens originating from the United Arabian Emirates excavated from archaeological sites (Al-Buhais, Umm an-Nar, Al-Sufouh, and Tell Abraq) were used to study the genetic profile of the mtDNA. The sequencing of mtDNA showed an unstructured camel population in North Africa and Asia. These findings indicated the absence of phylogeographic patterns reflecting the movements and the trading in the selected countries. They supposed that the coastal southeast Arabian Peninsula was a possible place of domestication and suggested a single domestication scenario with recurrent introgression from wild into the early domestic dromedaries (Almathen et al., 2016; Burger, 2016). In another study, two main maternal lineages related to the Middle-East and Eastern Africa were found in Tunisia after the comparison of the Tunisian camel mtDNA haplotypes and those available in DNA database (Nouaïria et al., 2018). Also, establishment of sub-groups was not possible because of a non-sufficient genetic structuring and nucleotide divergence.

### Single Nucleotide Polymorphism

SNPs are modifications of a single base pair that occur approximately every 1,000 bps in the mammalian genome (Riva and Kone, 2002). They are present on nuclear DNA and on mtDNA (Petkovski et al., 2005). This type of polymorphism is very abundant and uniformly distributed in the genome and constitutes the largest source of genetic polymorphism. These variations have been identified during different programs of genome sequencing known as “Expressed Sequence Tag programs” (Lee et al., 2006). Once obtained, these markers can be very helpful because of their easy to use and their reproducibility. They have been used in many studies on genetic diversity and relevant phenotypic traits for other species but have not yet been widely used in dromedaries. Recent studies are starting to use them to evaluate gene diversity at the level of specific genes (Lado et al., 2020a).

The development of SNP markers that cover the coding part of genome are also needed to understand the relationship between genetic and phenotypic variations in camels or other species. In these sites, SNPs are divided in synonymous, without any effect in the amino acid sequence and nonsynonymous affecting the amino acid sequence of protein (Sushma et al., 2014). In this study, the authors used direct ultra-high-throughput sequencing (RNA-Seq approach) for mapping and quantifying transcripts developed to analyze global gene expression in heart and kidney of *C. dromedarius* and C. *bactrianus* for the identification of gene and identifying polymorphism in camel at nucleotide level (SNPs). They identified 24 and 10 nonsynonymous SNPs in *C. dromedarius* in the heart and the kidney, respectively. In another study, Abd El-Aziem et al. (2015) detected one SNP (C/T) in Growth Hormone gene among five Egyptian camel breeds. They have concluded that this SNP can be used as a marker for the genetic diversity between the camel breeds. The allele C is related to higher growth rate and can be used in marker assisted selection for the enhancement of growth rate should it be needed in camel breeding program.

In another context, Ruiz et al., (2015) developed a diagnostic panel of SNPs to identify the hybridization patterns in camels with uncertain origins, to support hybrid breeding management and to detect potential rare dromedary introgression in the remaining wild Bactrian camels in Mongolia and China. Recently, in a genome-wide association study, Bitaraf Sani et al. (2021), using genotyping by sequencing, were able to identify 99 SNP markers that could be associated with important traits to improve camel breeding, namely birth, weight, daily gain and body weight.

Lado et al. (2020b), sequenced 22,721 SNP markers in order to understand how the dromedary populations history and the environmental adaptation could be influenced by human-induced migration patterns and historic demographic changes. The use of these molecular markers helped to understand the routes of domestication and how genetic diversity was built through the centuries. A genetic mixture within continental populations between Asia and Africa was detected.

In terms of genetic studies in livestock, SNPs have three main advantages over microsatellites: a more precise estimates of population-level diversity, a higher power to identify groups in clustering methods, and an ability to consider local adaptation (Zimmerman et al., 2020). These advantages offer better opportunities of using SNP in parentage control, GWAS for traits of economic interest and the diagnosis of genetic diseases by developing specific ships for dromedary camel.

## Dromedary Camel Parentage Control

In the biannual conference of the International Society for Animal Genetics, the Camel Comparison Test (CCT) for the laboratory developing parentage test for dromedary was discussed in a workshop named “Applied Genetics and Genomics in Other Species of Economic Interest” since 2016. For the parentage control, a minimum of eight microsatellites markers are needed (LCA19, LCA37, LCA56, LCA65, LCA66, LCA8, YWLL29, YWLL44) and are named Core panel in the CCT. For this test in 2016, twenty-four samples and one reference sample were submitted to all the laboratories participating in the CCT. A deadline was given and the participants were asked to send their results for the core panel which would be considered in the ranking system for an accreditation. It was possible to add nine other microsatellite markers in the back-up Panel (LCA24, LCA77, LCA99, LGU49, VOLP3, VOLP32, VOLP59, YWLL08, YWLL36) and 30 other microsatellites can be used also to compare results between participating laboratories. In the last CCT, the overall marker concordance among laboratories was good (>95%) for six of the eight markers in the Core Panel. Only two markers showed a lower concordance: LCA37 (86%) because of missed alleles and wrong allele binning and YWL44 (87%) because of a missed allele.

Different studies tried to use different panels of microsatellites to evaluate the exclusion power for their dromedary populations. In 2005, Nolte et al. used 16 microsatellites for the evaluation of genetic diversity and for parentage analysis in cattle. Five calves were compared to their known parents and an unrelated male. It was successful in all cases. Only one mismatch at the VOLP08 locus was observed in one calf. They conclude that this mismatch is possibly due to a mutation or a laboratory typing error. Mariasegaram et al. (2002) identified eight dromedary specific microsatellites as described previously and used them in racing dromedary camels for the evaluation of the probability of exclusion for a future use in parentage control. The total PE was high and estimated at 0.992. In Australia, Spencer et al. (2010) conducted a study to evaluate and apply microsatellite multiplexes to develop a parentage control for racing dromedaries. They randomly sampled dromedaries in three geographically separated regions. 17 loci from 700 unrelated dromedary samples of both sexes, including individuals from Australia (*n* = 620), United Arab Emirates (*n* = 53) and Africa (*n* = 16) were used to build a database of unrelated adults. The 17 microsatellites were separated in three multiplex reactions and a high probability of parentage exclusion (PE = 0.9999) was found. PE is an index allowing quantification of the percentage of incorrect detected affiliations. They conclude that this multiplex system clearly demonstrates the importance of DNA testing to ensure accurate identification and allocation of parentage in reproductive centers.

In Morocco, PE and the probability of identity (PI: probability to take hazardly two individuals with the same genotype) were determinated for five camel populations by using seven microsatellites markers (VOLP03 YWLL44, YWLL59, CVRL01, CVRL05, CVRL06 and CVRL07. PE varied from 95 to 97% with one parent and was higher than 99.99% with two parents for the five populations. The PI of individual camels varied between 1/8.10^–6^ and 1/55.10^–6^ (Piro et al., 2011). They noticed that, among the five studied populations, the CVRL1 followed by VOLP3 loci were the most effective loci to exclude the false parents, while YWLL44 and YWLL59 loci present the lowest PE and PI.

In Tunisia, Nouaïria et al. (2015) evaluated twenty microsatellites to develop a microsatellite panel for parentage control in 130 dromedary camels. They concluded that a minimum of 12 microsatellites are needed to obtain a PE higher than 99% when one parent is unknown parent and 98% when both parents are unknown in dromedary camel.

With the actual development of new molecular biology techniques and with regard to several other species, it will probably be obvious that the establishment of parentage control using SNPs will soon be proposed by ISAG. Therefore, studies to choose the most informative SNPs on this subject should be carried out.

## Genetic Structure of Different Breeds Around the World

Studies were conducted around the world on the genetic characterization and diversity of dromedary camels using microsatellites or SNPs (Lado et al., 2020b). Each study used a set of microsatellite markers to identify genetic difference between dromedary camels from different regions, in a specific country or through comparing breeds from different countries or continents. These different studies showed that it was quite difficult to establish a significant genetic differentiation between different types or breeds living in different regions and even with a different phenotype and most of the results suggested the probable existence of relatively less genetic variation in dromedary camels and that morphologic and regional distribution criterions are not enough for the dromedary camel classification or characterization (Jianlin et al., 2000; Mburu et al., 2003; Nolte et al., 2005; Vijh et al., 2007; Schulz et al., 2010; Ould Ahmed et al., 2010; Spencer and Woolnough, 2010; Piro et al., 2011; Mahrous et al., 2011; Mahmoud et al., 2012; Sushma et al., 2014; Sushma et al.,2015; Eltanany et al., 2015; Cherifi et al., 2017; Piro et al., 2018; Legesse et al., 2018; Tanveer et al., 2021). Not all the microsatellites markers used were polymorphic and the following markers showed a high level of polymorphism: CVRL01, CVRL07, CMS16, CMS50, CMS121, LCA56, LCA63, LCA65, LCA66, LCA70, VOLP03, VOLP10, VOLP32, VOLP50, VOLP55, VOLP67, YWLL08, YWLL09 (Jianlin et al., 2000; Nolte et al., 2005; Tyagi et al., 2017).

A higher level of cross-breeding or a gain of genetic variation following genetic drift subsequent to migration from one area to another, and traditional herding practices and dromedary camel particular history of domestication likely occurring from a bottlenecked and geographically confined wild progenitor can explain the poor genetic variation (Mahmoud et al., 2012; Cherifi et al., 2017; Piro et al., 2018). Hashim et al., (2014) indicated also that there was a relationship between the genetic makeup and geographical distributions and also between the genetic makeup and the phenotypic characteristic. Moreover, it is worth mentioning that most camelid populations are named as breeds after their tribal affiliation or the geographic location where they are found (Rosati et al., 2005). Only one study reported the existence of genetic variation amongst the four camel breeds studied in Libya (Bakory, 2012) and Banerjee et al. (2012) compared six Indian camel populations and showed a genetic differentiation among themselves due to selection pressure and breeding for specific economic traits and that the camel populations of India and South Africa are very well differentiated. Mehta, (2014) used more microsatellites (40) to analyze the genetic and demographic bottleneck of Indian camel breeds (Bikaneri, Jaisalmeri, Kachchhi and Mewari camel breeds) and they indicated that allelic polymorphism was observed only in 20 loci and a higher genetic variation was observed in most numerous Bikaneri breed. In comparison with the other three Indian dromedary breeds, the Mewari camels had relatively higher genetic distance from other breeds. These authors concluded that their results showed a demographic bottleneck in the four Indian dromedary populations and an appropriate conservation and improvement program is necessary.

The main studies using microsatellites and undertaken in several countries are reported in table 2, with emphasis on the number of markers used, the species and the regions studied. Recently, Lado et al., (2020b) used 22K SNPS markers for the evaluation of genetic diversity of African and Asian dromedary camels and showed also the existence of moderate genome-wide diversity and a low population structure.

**TABLE 2:** Main studies using microsatellites realized in dromedary camels for the evaluation of genetic diversity.

Species studied	Regions or countries	Number of microsatellites used	References
Dromedary camel and Bactrian camels	Kenya and China	20	Jianlin et al. (2000)
Dromedary camel and Bactrian camels	Kenya, Pakistan, United Arab Emirates, Saudi Arabia and China	14	Mburu et al. (2003)
Dromedary camel	India	6	Gautam et al. (2004)
Alpaca and Dromedary camel	South Africa, Namibia and Botswana	16	Nolte et al. (2005)
Dromedary camel	India	16	Mehta and Sahani, (2007)
Dromedary camel	India	23	Vijh et al. (2007)
Dromedary camel	Australia and africa	28	Spencer and Woolnough (2010)
Dromedary camel	Canarias, Algeria, Kenya, Saudi Arabia, UAE and Pakistan	13	Schulz et al. (2010)
Dromedary camel	Tunisia	6	Ould Ahmed et al. (2010)
Dromedary camel	Egypte	3	Mahrous et al. (2011)
Dromedary camel	Morocco	7	Piro et al. (2011)
Dromedary camel	Saudi Arabia	15	Mahmoud et al. (2012)
Dromedary camel	Libya	6	Bakory (2012)
Dromedary camel	India	12	Banerjee et al. (2012)
Dromedary camel	Sudan, Qatar, Somalia and Chad	25	Hashim et al. (2014)
Dromedary camel	India	40	Mehta (2014)
Dromedary camel	India	29	Sushma et al, (2014)
Dromedary camel	Tunisia	7	Nouaïria et al. (2015)
Dromedary camel	Sudan	12	Eltanany et al. (2015)
Dromedary camel	India	25	Sushma et al. (2015)
Dromedary camel	Saudi Arabia	18	Almathen et al. (2016)
Dromedary camel	Algeria and Egypt	20	Cherifi et al. (2017)
Dromedary camel	India	11	Tyagi et al. (2017)
Dromedary camel	Morocco	19	Piro et al. (2018)
Dromedary camel	Ethiopia	6	Legesse et al. (2018)
Dromedary camel	Pakistan	12	Tanveer et al. (2021)

## Conclusion

The dromedary camel is a species of great zootechnical interest in several countries. Generally, camel genetic characterization studies have mainly been completed using microsatellite markers. However, to better characterize certain production interesting traits, other techniques using whole genome sequencing and SNPs are necessary. Currently, these techniques are under investigation to assess relation with some phenotypic trait. These studies will allow better understanding of this magnificent exotic species, better conservation of the different breeds and types of dromedary and would certainly provide better selection alternatives for the breeders.
